# Lateral unicompartmental knee arthroplasty (UKA) showed a lower risk of failure compared to medial unicompartmental knee arthroplasty in the Register of Prosthetic Orthopedic Implants (RIPO)

**DOI:** 10.1007/s00402-022-04631-x

**Published:** 2022-09-25

**Authors:** Domenico Alesi, Barbara Bordini, Stefano Fratini, Cristina Ancarani, Piero Agostinone, Alberto Grassi, Giulio Maria Marcheggiani Muccioli, Marco Viceconti, Stefano Zaffagnini

**Affiliations:** 1 Clinica Ortopedica e Traumatologica II, IRCCS Istituto Ortopedico Rizzoli, Via Pupilli 1, 40136 Bologna, BO Italy; 2 Laboratorio di Tecnologia Medica, IRCCS Istituto Ortopedico Rizzoli, Via di Barbiano 1/10, 40136 Bologna, BO Italy

**Keywords:** Lateral unicompartimental knee arthroplasty, Modes of failure, Revision rate, Arthroplasty registry, Registry study

## Abstract

**Introduction:**

The present study aimed to investigate differences in survivorship between medial and lateral unicompartmental knee arthroplasty (UKA) by analyzing the data of an Italian regional registry. The hypothesis was that, according to recent literature, lateral implants have comparable survivorship with regard to the medial implants.

**Materials and methods:**

The Register of Orthopaedic Prosthetic Implants (RIPO) of Emilia-Romagna (Italy) database was searched for all UKAs between July 1, 2000, and December 31, 2019. For both cohorts, subject demographics and reasons for revision were presented as a percentage of the total cohort. Kaplan–Meier survivorship analysis was performed using revision of any component as the endpoint and survival times of unrevised UKAs taken as the last observation date (December 31, 2019, or date of death).

**Results:**

Patients living outside the region and symmetrical implants (which do not allow the compartment operated to be traced) were excluded. 5571 UKAs implanted on 5172 patients (5215 medial UKAs and 356 lateral UKAs) were included in the study. The survivorship analysis revealed 13 failures out of 356 lateral UKAs (3.7%) at a mean follow-up of 6.3 years and 495 failures out of 5215 medial UKAs (9.5%) at a mean follow-up of 6.7 years. The medial UKAs had a significantly higher risk of failure, with a Hazard Ratio of 2.6 (CI 95% 1.6–4.8; *p* < 0.001), adjusted for age, gender, weight, and mobility of the insert. Both the groups revealed a good survival rate, with 95.2% of lateral implants and 87.5% of medial implants still in situ at 10 years of follow-up.

**Conclusions:**

Lateral UKA is a safe procedure showing longer survivorship than medial UKAs (95.2% and 87.5% at 10 years, respectively) in the present study.

**Level of evidence:**

Level 3, therapeutic study.

## Introduction

Unicompartimental knee arthroplasty (UKA) is a viable alternative to total knee arthroplasty (TKA) in patients with both medial or lateral isolated knee osteoarthritis [1]. UKA has several potential advantages over TKA, which include improved postoperative outcomes, a less invasive procedure, preservation of bone stock and ligamentous structures, improved proprioception, earlier return to activities, shorter hospital stay, and generally a higher patient satisfaction with fewer complications [2–6].

Fewer lateral UKAs are implanted each year in comparison to medial ones. This is mainly due to the higher prevalence of isolated medial knee osteoarthritis, with studies reporting that medial UKAs are performed ten times more often than lateral UKA [7]. Therefore, studies analyzing lateral UKA have smaller sample sizes and the literature provides limited information; moreover, registry studies often analyze the survival of UKA, not separating medial and lateral implants and are mainly focused on comparing UKA and TKA, regardless of laterality [8, 9].

Medial and lateral compartments should be instead considered separately, given the different anatomy, biomechanics, and weight loads [10]. The convexity of the lateral tibial plateau and the C-shaped lateral meniscus provide wider mobility to the lateral compartment than the medial side. Therefore, the lateral femoral condyle articulates with the posterior tibial plateau in deep flexion. Moreover, the femoral rollback is greater, and the screw-home mechanism is far more significant on the lateral side [11]. These differences lead to considering that UKA ideally requires different positioning and implants for medial and lateral compartments. This behavior could be appreciated in the early mobile-bearing UKA design where the flat tibial component, similar to the medial anatomy, had a higher risk of bearing dislocation when implanted laterally [12].

Medial implants were historically considered at a lower risk of failure, for the higher complexity of lateral compartment biomechanics and the scarcity of studies investigating lateral UKAs designs and positioning [13]. Only few case series analyzed differences in outcomes and survivorship between lateral and medial UKA, and no consensus exists about the topic, with some series reporting inferior survivorship for lateral implants and others highlighting worse function for medial UKAs [14]. Moreover, a recent meta-analysis by Seung-Beom Han et al. found that short- to mid-term and long-term survival did not significantly differ between medial and lateral UKAs [13].

The Register of Prosthetic Orthopedic Implants (RIPO) of Emilia-Romagna is a large regional implant registry in Italy, firstly introduced at Istituto Ortopedico Rizzoli in 1990 and later expanded to the whole Emilia-Romagna region since 2000, including more than 4 million people [15]. The present study aimed to investigate differences in survivorship between medial and lateral UKA by analyzing the data of this regional Italian registry. The hypothesis was that, according to recent literature, lateral implants have comparable survivorship to the medial implants.

## Methods

The Register of Orthopaedic Prosthetic Implants (RIPO) database was searched for all UKAs between July 1, 2000, and December 31, 2019. The RIPO was established at the Istituto Ortopedico Rizzoli in Bologna and collects data related to hip, knee, and shoulder arthroplasty procedures (primary and revision surgeries) performed in the Emilia-Romagna region (Italy) since 2000.

UKAs were excluded if they were implanted in patients outside the Emilia-Romagna region to minimize bias due to loss to follow-up. For residents of the Emilia-Romagna region, any treatment received in other regions of Italy is billed back to the Emilia-Romagna region and therefore captured in the registry.

For both cohorts, subject demographics and reasons for revision were presented as a percentage of the total cohort. Patient age and weight were compared using a *t*-test, side operated, gender and mobility of the insert were compared using Fisher test, while indication for surgery, femur and insert material, and fixation were compared using chi-square analysis. Differences between the groups were considered statistically significant if the *p* value was less than 0.05. Kaplan–Meier survivorship analysis was performed using revision of any component as the endpoint and survival times of unrevised UKAs taken as the last observation date (December 31, 2019, or date of death). The Wilcoxon Test test was used to compare survivorship between the two groups. The Cox multiple regression model for analyzing survival data was considered. The proportionality hazards assumption was tested by the Schoenfeld residual method.

The statistical analysis was performed using SPSS 14.0 for Windows, version 14.0.1 (SPSS Inc, Chicago, IL, USA) and JMP, Version 12.0.1 (SAS Institute Inc., Cary, NC, 1989–2007).

Ethics approval was not necessary as the data were collected from a registry in Italy that collects data as standard practice on all patients in their region. Additionally, all data were collected and analyzed in a de-identified format that protects patient privacy.

## Results

The present registry analysis collected data from 7568 UKAs performed between July 2000 and December 2019 on patients resident in the Emilia-Romagna region. Patients living outside the region were excluded to avoid the bias of possible lost follow-up. After excluding 1997 symmetrical implants which, as they could be used indiscriminately for the medial and lateral femoro-tibial compartment, did not allow the compartment operated to be traced, 5571 UKAs implanted on 5172 patients were included in the study. 5215 medial UKAs and 356 lateral UKAs were present in this cohort.

Analyzing the total number of procedures performed by year of surgery and compartment treated, from 2000 to 2019, lateral UKAs account for 6.4% and medial UKAs for 93.6% of the total, with a progressive increase in the total number of patients treated over the years.The demographics of the two groups are shown in Table 1.

**Table 1:** Demographic data of the study cohort

Descriptive statistics	Lateral	Medial	Comparison between groups
*No of implants (%)*	356 (6.4%)	5215 (93.6%)	
*No of patients*	343	4829	
Side operated			Signif. Fisher test *p* < 0.001
Right (%)	244 (68.5%)	2698 (51.7%)	
Left (%)	112 (31.5%)	2517 (48.3%)	
*Gender*			Signif. Fisher test *p* < 0.01
Female (%)	256 (71.9%)	3389 (65.0%)	
Male (%)	100 (28.1%)	1826 (35.0%)	
*Mean age (standard deviation) (range)*	65.5 (SD 10.5) (24–90)	67.0 (SD 8.7) (28–92)	Signif. *T*-test *p* < 0.01
Weight*	74.6 (11.5)	77.3 (13.0)	Signif. *T*-test *p* < 0.001
Average (standard dev.)			
% > 80 kg	24.3%	34.3%	
Diagnosis**			Signif. Chi-square test *p* < 0.001
Primary arthritis (%)	82.6%	82.3%	
Deformity (%)	6.8%	8.6%	
Necrosis of the cond. (%)	4.6%	6.1%	
Post-traumatic and Sequelae of fracture (%)	4.6%	1.9%	
Other (%)	1.4%	1.1%	
*Mobility of the insert*			Signif. Fisher test *p* < 0.001
Fixed (%)	334 (93.8%)	4176 (80.1%)	
Mobile (%)	22 (6.2%)	1039 (19.9%)	
*Insert material*	98.3	96.2	NS Chi-square test *p* = 0.09
Standard poly(%)	1.7	2.8	
Crosslinked poly(%)	-	1.0	
Cross.antiox. poly (%)			
*Femur material*			NS Chi-square test *p* = 0.50
Crco(%)	75.3%	73.1%	
Ceramicised zirconium (oxinium) (%)	24.7%	26.6%	
Ceramicised cr-co(%)	–	0.4%	
*Prosthesis fixation*			NS Chi-square test *p* = 0.61
Cemented(%)	98.3%	97.6%	
Cementless(%)	1.4%	2.2%	
Hybrid (%)	0.3%	0.2%	

Statistically significant differences were identified for knee laterality, gender, age, weight, diagnosis, and insert type. The main causes of UKA implant were primary osteoarthritis followed by axial malalignment and osteonecrosis.

The survivorship analysis revealed 13 failures out of 356 lateral UKAs (3.7%) at a mean follow-up of 6.3 years and 495 failures out of 5215 medial UKAs (9.5%) at mean follow-up of 6.7 years. The medial UKA had a significantly higher risk of failure, with a Hazard Ratio of 2.6 (CI 95% 1.6–4.8; *p* < 0.001), adjusted for age, gender, weight, and mobility of the insert.

**Fig. 1:**
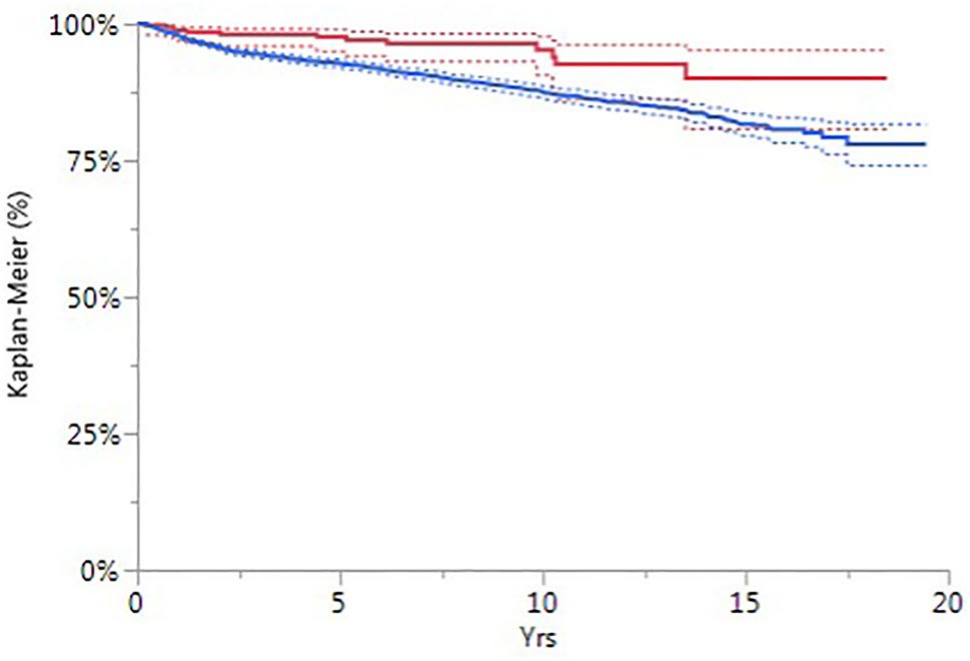
Kaplan–Meier diagram showing the survivorship per year for lateral and medial UKA. The authors have no financial or proprietary interests in any material discussed in this article

The survivorship per year for each group is reported in Table 2 and graphically represented in the Kaplan–Meier diagram in Fig. 1. Both the groups revealed a good survival rate, with 95.2% of lateral implants and 87.5% of medial implants still in situ at 10 years of follow-up.

**Table 2:** Survivorship per year for each group

Condyle	% survival (Confidence interval 95%)					
	1 Year	3 Years	5 Years	7 Years	10 Years	13 Years
*Lateral*	98.8 (96.8–99.5)	98.1 (95.8–99.1)	97.6 (95.0–98.9)	96.4 (93.1–98.2)	95.2 (90.6–97.7)	92.7 (86.2–96.2)
Prostheses at risk	313	238	179	135	75	41
*Medial*	97.9 (97.5–98.3)	94.5 (93.8–95.1)	92.7 (91.9–93.5)	90.8 (89.8–91.7)	87.5 (86.3–88.7)	84.8 (83.2–86.2)
Prostheses at risk	4647	3616	2923	2275	1371	676

The main causes of revision were, as follows, aseptic loosening (23.1% and 38.0% for lateral and medial UKAs, respectively), pain without loosening (23.1% and 22.6%), and septic loosening (6.5% for medial UKAs). In the present cohort, no case of septic loosening was reported for the lateral UKAs. The detailed information about causes of revision was reported in Table 3.

**Table 3:** Revision causes

Cause of revision	Lateral			Medial		
	Rate	Percen-age	% distribut. of failure causes	Rate	Percen-tage	% distribut. of failure causes
Total aseptic loosening	3/356	0.8	23.1	188/5215	3.6	38.0
Pain without loosening	3/356	0.8	23.1	112/5215	2.1	22.6
Tibial aseptic loosening	*–*	*–*	*–*	72/5215	1.4	14.5
Septic loosening	*–*	-	*–*	32/5215	0.6	6.5
Femoral aseptic loosening	*–*	*–*	*–*	16/5215	0.3	3.2
Dislocation	*–*	*–*	*–*	12/5215	0.2	2.4
Insert wear	*–*	*–*	*–*	12/5215	0.2	2.4
Breakage of prosthesis	1/356	0.3	7.7	10/5215	0.2	2.0
Periprosthetic bone fracture	2/356	0.6	15.4	4/5215	0.1	0.8
Instability	*–*	*–*	*–*	2/5215	0.0	0.4
Other	*–*	*–*	*–*	8/5215	0.2	1.6
Unknown	4/356	1.1	30.8	27/5215	0.5	5.5
Total	13/356	3.7	100.0	495/5215	9.5	100.0

3 patients with prior medial UKA implanted subsequently underwent lateral UKA, and two patients underwent patellofemoral implant after medial UKA; although the reoperations, these patients were not considered failures for the purposes of the study.

## Discussion

The main finding of the present study was that the survival rate at 10 years of follow-up was significantly higher for lateral UKAs with respect to medial UKAs. Interestingly, the literature provides conflicting results on the topic. Articles that evaluated the survival of isolated lateral UKAs reported satisfactory results. Deroche et al. showed high survivorship at long term (mean follow-up of 17 years) for lateral UKAs, with a survival rate of 82.1% at 15 years [16]. The same author also participated in a multicenter study that showed very good overall survival rate (85.4% at 10 years among 268 lateral UKAs), considering the long follow-up period and older techniques used in the early years, such as anterior tibial tubercle osteotomies [17]. This can explain the lower results in comparison with the series presented in the current study, which showed a better survival rate of 92.7% for lateral UKAs at 13 years of follow-up. Other studies, however, show no difference among medial and lateral UKA survivorship. A recent metanalysis by Han et al. showed no significant differences in the survival of lateral UKAs compared to medial UKAs [13]. A systematic review by Van der List et. al. reported no significative difference in survivorship and revision rates, however pointing out overall lower implant survivorship in registry studies (90.5% vs 84.1% combined UKA survivorship at 10 years for cohort and registry studies respectively) [18]. According to the authors, this latter statement can be explained with cohort studies being often performed in high volume centers, whereas registry-based studies also report low-volume center outcomes. In studies comparing outcomes in high-volume centers with low-volume centers, better results can be found in the first ones [19, 20].

No recent studies can be found showing higher survivorship for medial UKAs compared to lateral UKAs. Those results could be explained by the different behavior of the lateral knee side compared to the medial. During deep flexion, the medial compartment stays relatively static on the anteroposterior (AP) plane, with 1.5 mm of translation, while the lateral side has an intrinsic instability with a greater degree of freedom and translations that can vary between 9 and 15 mm [21, 22]. Moreover, the lateral compartment is less involved in weight transmission, both during stance and walking phase [23, 24].

Historically, the reasons for worse survival of lateral UKAs were the more complex surgical technique and the poor knowledge of lateral knee osteoarthritis and kinematics, which led surgeons having limited experience in implanting lateral UKAs. The surgeon learning curve for the lateral side is steeper than the one for the medial, and, all factors considered, there is an essential difference among the same procedures performed by different surgeons. One of the factors that justify the controversies found in literature is the surgeon experience, given that the number of lateral UKAs performed plays an important factor in optimizing outcomes and survivorship.

Many authors underline, when explaining potential causes of failure of lateral UKAs, how it is important to perform a different surgical technique in comparison to the medial side [17, 18, 25], minimizing tibial cuts and internally rotating the sagittal cuts to avoid impingement between the femoral component and the tibial spine in extension [26, 27]. Moreover, the femoral component should be placed as lateral as possible to avoid unwanted translations in extension induced by the “screw-home” mechanism, and the tibial slope should be neutral to respect the normal anatomy, which again differs from the medial compartment [28].

Another factor that could influence the survivorship of lateral UKAs is the more recent design development of such implants regarding the medial side, with the latter available on the market for a more extended period. However, additional time will be needed to prove it statistically [18].

This study has some limitations, mainly because the data included in the RIPO registry are standardized but not complete, no information about the subjective outcomes are collected, and it only includes cases from the region Emilia-Romagna. However, the RIPO records UKA implants specificities differently from other registries, allowing us to discriminate, for example, from medial to lateral prostheses. Lastly, as per every registry study, one of the most valuable features is the vast number of procedures evaluated, which is very difficult to match in trials and case series.

Given actual evidence and data collected from the present large cohort, lateral UKA is a safe procedure and can be confidently proposed to patients with isolated lateral knee osteoarthritis and intact ligaments.

## Conclusions

Lateral UKA is a safe procedure showing longer survivorship than medial UKAs (95,2% and 87,5% at 10 years, respectively) in the present study and can be confidently proposed to the patients with isolated lateral knee osteoarthritis and intact ligaments.
